# The Unusual Suspect: Gadobenate-Dimeglumine Induced Kounis Syndrome

**DOI:** 10.51894/001c.8999

**Published:** 2019-07-01

**Authors:** Faiza Choudhry, Michael Fackler, Mithil Patel, Vijay Patel, Jelena Arnautovic

**Affiliations:** 1 Department of Cardiovascular Medicine Ascension Macomb-Oakland Hospital; 2 Department of Cardiovascular Medicine Henry Ford Macomb Hospital

**Keywords:** gadobenate dimeglumine, acute coronary syndrome, allergic angina, kounis syndrome

## Abstract

A myocardial bridge has traditionally been considered a benign condition characterized by an atypical intramyocardial route of a segment of one of the major coronary arteries. However, the clinical complications of myocardial bridges can be dangerous. These potential complications include acute coronary syndromes, arrhythmias, ventricular dysfunction, and sudden death. Myocardial bridges are suspected to be adjuvant of Kounis syndrome, which is defined as an acute coronary syndrome caused by an allergic reaction. Due to high epidemiologic prevalence, clinical suspicion of a myocardial bridge should be considered in atypical and typical presentations of chest pain, especially in patients with low-risk factors for atherosclerotic disease. A male in their late 30’s presented with non-ST elevation myocardial infarction suspected to be secondary to Kounis syndrome after gadobenate dimeglumine contrast media was used for an imaging study. His clinical presentation was further complicated when he was found to have a mid-left anterior descending coronary artery myocardial bridge.

## Introduction:

Kounis syndrome is defined as an acute coronary syndrome caused by allergic, hypersensitivity, and anaphylactoid reaction.^1^ It has also been referred to as an “allergic angina syndrome.” There are three variants of Kounis syndrome. The type I variant involves acute coronary syndrome caused by coronary artery vasospasm in patients without pre-existing coronary artery disease and no elevation in cardiac enzymes. The type II variant involves coronary vasospasm leading to plaque erosion or rupture in patients with quiescent pre-existing coronary artery disease leading to acute myocardial infarction. The type III variant involves patients with coronary artery stent thrombosis in which thrombus specimens demonstrates the presence of mast cells and eosinophils.^2^

Several kinds of food, drugs, and environmental exposures have been listed as triggers of Kounis syndrome. While multiple intravenous contrast media have been known to cause Kounis syndrome, there is no documented report of gadobenate dimeglumine causing such a reaction according to our review of literature. Gadobenate dimeglumine is used worldwide in magnetic resonance imaging and off-label for cardiac magnetic resonance imaging. Its side effects include chronic kidney disease as well as anaphylactic reactions that can lead to cardiac arrest in rare instances.^3,4^ The incidence of coronary vasospasm and myocardial infarction following administration of gadolinium contrast is unclear, and the following case depicts such a scenario.

## Case Description:

**Figure 1:**
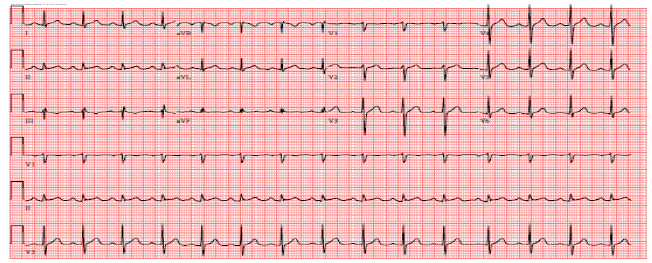
Admission electrocardiogram demonstrated normal sinus rhythm with no acute ST deviations

A male in his late 30’s with no significant past medical history was evaluated for a complaint of episodic double vision with magnetic resonance imaging of the brain by using gadobenate dimeglumine contrast medium. Following gadobenate dimeglumine administration, the patient developed urticaria, chest tightness, and laryngospasm. He was treated with epinephrine, intravenous steroids, and antihistamines in the emergency department with relief of symptoms and discharged home with oral steroids and antihistamines. He returned 16 hours later with severe sub-sternal chest pain. Initial vital signs were stable with no clinical evidence of overt heart failure. Troponins were elevated and eventually peaked at 6.65 ng/mL with no electrocardiographic evidence of ischemia (Figure 1).

**Figure 2 A-B:**
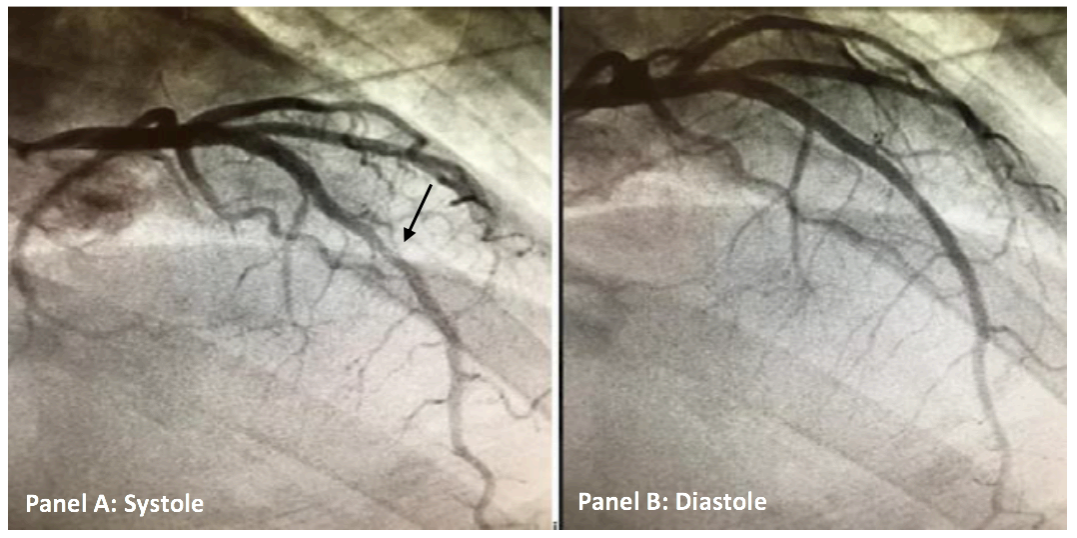
Coronary angiogram of the left anterior descending coronary artery (LAD) in both systole (A) and diastole (B). There is a normal appearance of the LAD during diastole and marked compression of middle segment of LAD (black arrow) noted during systole due to myocardial bridge.

The patient was admitted to the hospital for non-ST elevation myocardial infarction and guideline directed medical therapy with anti-coagulation using intravenous heparin and anti-platelet therapy using aspirin was instituted. Anti-coagulants are a class of medications commonly referred to as blood thinners. They are used to prevent coagulation of blood and to prolong the clotting time.^5^ Coronary angiography was performed as the patient was having persistent chest pain despite optimal medical therapy and supportive care including nitrates and anti-anginal agents. Nitrates are a class of medication commonly used during acute coronary syndromes for symptomatic relief. Coronary angiography revealed normal coronary arteries with myocardial bridging of the mid-left anterior descending artery (Figure 2A-B). Since patient coronary arteries were unremarkable for any stenosis per angiography, intravascular ultrasound technique was not utilized. Intravascular ultrasound is a type of imaging used frequently to assess for atheromatous plaque burden in coronary arteries.^6^

Echocardiography demonstrated a normal ejection fraction with no regional wall motion abnormalities and no pericardial effusion. One episode of non-sustained ventricular tachycardia was noted during hospitalization. Chest pain and troponin elevation eventually resolved with volume expansion, intravenous steroids, and antihistamines. The patient was discharged home after three days with a steroid taper, amlodipine, and metoprolol with subsequent uneventful ambulatory follow-up.

## Discussion:

This case describes a coronary artery spasm of a myocardial bridge due to acute allergic insult secondary to gadobenate dimeglumine leading to endothelial dysfunction. The absence of atherosclerotic lesions on coronary angiography suggests allergic angina as the main pathophysiologic mechanism in this case. This represents a type I variant of Kounis syndrome, although it was atypical in the delayed response. To our knowledge, this is the first report documenting the association between gadobenate-dimeglumine and Kounis syndrome in English literature.

The introduction of an allergen, gadobenate dimeglumine in this case, can induce degranulation of mast cells and activation of several pro-thrombotic and vasoconstrictor mediators, including histamine, prostaglandins, and leukotrienes. The release of these mediators results in generalized acute allergic reaction. Gadobenate dimeglumine is noted to have the highest incidence of immediate hypersensitivity reactions.^7,8^ Male patients tend to have more severe reactions. These acute allergic reactions are rarely followed by acute coronary syndrome. Mast cells are found in all tissues of the human body, including the heart and blood vessels. They are noted to be present in artherosclerotic lesions during acute coronary syndrome triggered by allergic reaction as well as in normal coronary arteries during vasospasms.^9^ Cardiac mast cells respond to not only IgE-mediated stimuli but can also be activated by radiocontrast media. They release chymase and renin upon liberation which further activates the renin-angiotensin system and promotes local secretion of noradrenaline with subsequent stimulation of the adrenergic system.^10^ This may explain cardiac arrhythmias, coronary vasospasms, myocardial infarction, and sudden cardiac death associated with anaphylaxis. The administration of epinephrine for the treatment of our patient’s anaphylactic reaction further provoked adrenergic activity resulting in a coronary spasm of the mid-left anterior descending artery myocardial bridge.

Myocardial bridging, which has traditionally been considered a benign condition, placed our patient at an increased risk for coronary vasospasm. The prevalence and pathophysiology of myocardial bridges is not fully known. It is estimated to occur in one-fourth of adults and is usually an incidental finding on angiography or autopsy.^11^ Its prevalence is noted to be as high as 40-80% on autopsy, but functional myocardial bridging is observed less commonly on angiography. The majority of myocardial bridges occur in the left anterior descending coronary artery. The presence of myocardial bridging on the mid-left anterior descending artery increases the risk of coronary atherosclerosis in the segment proximal to the bridge by about five times secondary to the hemodynamic forces.^11^

Traditional coronary risk factors can further worsen the atherosclerotic burden in patients with myocardial bridging.^9^ Despite the low coronary risk profile of our patient, the accumulation of mast cells at the proximal segment of the LAD induced myocardial damage. Functional and anatomical alterations of the coronary vessel induced by myocardial bridging can lead to vasospasm at the mid-LAD.^12^ Some authors consider myocardial bridging as an adjuvant of Kounis syndrome. There have been two reported cases of the type I variant of Kounis syndrome leading to myocardial infarction in the setting of myocardial bridging.^9,12^ Hence, it can be concluded that a patient with myocardial bridging presents with a more severe presentation of Kounis syndrome.

## Conclusions:

Anaphylactic reactions to gadolinium-based contrast, such as coronary spasm as demonstrated in our case, are exceedingly rare. Prompt recognition of patients with increased risk of detrimental outcomes following an allergic reaction is necessary. Kounis syndrome should be considered in patients presenting with chest pain and a clinical presentation consistent with acute coronary syndrome who have recently been treated for allergic reaction, as an acute coronary syndrome presentation follows the anaphylactoid reaction. Kounis syndrome is not rare but is certainly underdiagnosed. It is also of paramount importance to treat Kounis syndrome appropriately.

Typical drugs such as morphine and beta blockers used for acute coronary syndrome should be avoided in patients with Kounis syndrome. Morphine can potentially stimulate histamine release and beta-blockers can potentiate coronary vasospasm due to an unopposed alpha-adrenergic response in these patients. Epinephrine should also be used with caution as it can potentially worsen coronary vasospasm and coronary ischemia.^1^ The focus of treatment should be the removal of offending allergens and supportive care. Prompt treatment of the allergic reaction can prevent further fatalities and adverse cardiac outcomes. This case illustrates the constant need to be vigilant about the side effects of frequently administered and typically benign agents such as gadolinium chelates. This is especially important in a patient population whose coronary arteries bridge the myocardium and predispose them to an increased risk for detrimental outcomes in response to allergic reactions.

### Conflict of Interest

The authors declare no conflict of interest.
